# Pregnancy planning does not interfere with child development in children aged from 11 to 23 months old*

**DOI:** 10.1590/1518-8345.5356.3506

**Published:** 2021-11-19

**Authors:** Katherine Solís-Cordero, Luciana Assis Couto, Luciane Simões Duarte, Ana Luiza Vilela Borges, Elizabeth Fujimori

**Affiliations:** 1 Universidade de São Paulo, Escola de Enfermagem, São Paulo, SP, Brazil.; 2 Scholarship holder at the Programa Institucional de Bolsas de Iniciação Científica/Conselho Nacional de Desenvolvimento Científico e Tecnológico (PIBIC/CNPq), Brazil.; 3 Secretaria de Estado da Saúde do Estado de São Paulo, Divisão de Doenças Crónicas não Transmissíveis, São Paulo, SP, Brazil.

**Keywords:** Child Development, Unplanned Pregnancy, Child Health, Women’s Health, Maternal and Child Health, Primary Care Nursing, Desarrollo Infantil, Embarazo no Planeado, Salud del Niño, Salud de la Mujer, Salud Materno-Infantil, Enfermería de Atención Primaria, Desenvolvimento Infantil, Gravidez Não Planejada, Saúde da Criança, Saúde da Mulher, Saúde Materno-Infantil, Enfermagem de Atenção Primária

## Abstract

**Objective:**

to analyze the correlation between child development and pregnancy planning and other associated aspects.

**Method:**

a cross-sectional study conducted with 125 mother-child dyads, the children aged from 11 to 23 months old and attending daycare centers located in socially disadvantaged areas. Child development according to domains was assessed using the Ages & Stages Questionnaire-BR and pregnancy planning was evaluated through the London Measure of Unplanned Pregnancy. The mothers were interviewed at their homes and non-parametric tests were used for data analysis.

**Results:**

17.6% of the pregnancies were unplanned, 24.8% were planned and 57.6% were ambivalent. Inadequate development in the different domains ranged from 21% to 40% and was not associated with pregnancy planning. However, the “communication” domain was associated with *Bolsa Família* and the “personal/social” and “communication” domains, with gender; while “personal/social”, “broad motor coordination” and “fine motor coordination” were domains related to the child’s age.

**Conclusion:**

no correlation between pregnancy planning and child development was observed; however, the low frequency of planned pregnancies and the high percentages of inadequate child development show the need to invest in the training of health professionals, both for contraceptive care and preconception health and for the promotion of child development, especially in socioeconomically disadvantaged contexts.

## Abstract (es)

**Objetivo:**

analizar la relación entre el desarrollo infantil y la planificación del embarazo y otros aspectos asociados.

**Método:**

estudio transversal realizado con 125 díadas madre e hijo de 11 a 23 meses, que concurren a guarderías infantiles ubicadas en zonas socialmente desfavorables. El desarrollo infantil de acuerdo con los dominios se evaluó mediante el *Ages & Stages Questionnaire-BR* y la planificación del embarazo mediante la *London Measure of Unplanned Pregnancy*. Las madres fueron entrevistadas en sus domicilios y se utilizaron pruebas no paramétricas para el análisis de los datos.

**Resultados:**

se registr*ó que* 17,6% de los embarazos no fue planificados, 24,8% fue planificado y 57,6% ambivalente. El desarrollo inadecuado en los diferentes dominios osciló entre el 21% y el 40% y no estaba asociado con la planificación del embarazo. Sin embargo, el dominio “comunicación” se asoció con Bolsa Familia; los dominios “personal/social” y “comunicación” con el sexo; mientras que los dominios “personal/social”, “coordinación motora gruesa” y “coordinación motora fina” estaban relacionados con la edad del niño.

**Conclusión:**

no se observó relación entre la planificación del embarazo y el desarrollo infantil, sin embargo, la baja frecuencia de embarazos planificados y los altos porcentajes de desarrollo infantil inadecuado demuestran que es necesario invertir en la capacitación de los profesionales de la salud, tanto para la atención sobre anticoncepción y salud preconcepcional, como para la promoción del desarrollo infantil, especialmente en contextos socioeconómicos desfavorables.

## Abstract (pt)

**Objetivo:**

analisar a relação entre desenvolvimento infantil e planejamento da gravidez e outros aspectos associados.

**Método:**

estudo transversal conduzido com 125 díades mãe-criança de 11 a 23 meses de idade, frequentadoras de creches localizadas em áreas socialmente desfavoráveis. O desenvolvimento infantil segundo domínios foi avaliado com aplicação do *Ages & Stages Questionnaire-BR* e o planejamento da gravidez por meio do *London Measure of Unplanned Pregnancy*. Mães foram entrevistadas nos domicílios e utilizou-se testes não paramétricos para análise dos dados.

**Resultados:**

verificou-se 17,6% de gravidez não planejada, 24,8% foram planejadas e 57,6% ambivalentes. O desenvolvimento inadequado nos diferentes domínios variou de 21-40% e não teve associação com o planejamento da gravidez. No entanto, o domínio “comunicação” associou-se com Bolsa Família; os domínios “pessoal/social” e “comunicação” com sexo; ao passo que “pessoal/social”, “coordenação motora ampla” e “coordenação motora fina” foram domínios relacionados com a idade da criança.

**Conclusão:**

não foi observada relação entre o planejamento da gravidez e o desenvolvimento infantil, porém, a baixa frequência de gestações planejadas e os elevados percentuais de inadequado desenvolvimento infantil mostram a necessidade de se investir na capacitação dos profissionais de saúde, tanto para a atenção em contracepção e saúde pré-concepcional, quanto para a promoção do desenvolvimento infantil, especialmente em contextos socioeconômicos desfavoráveis.

## Introduction

Child development involves physical growth, neurological maturation, and acquisition of cognitive and psychosocial functions^(1)^. In low- and middle-income countries, a high proportion of children under five years of age do not reach their development potential, impairing their learning ability and social and emotional skills, with negative impacts on future quality of life^(2)^.

Many variables have been associated with child development, such as social conditions^(3-4)^, maternal schooling and work^(5-6)^, the mother’s age^(7)^ and prenatal care^(8)^, in addition to variables inherent to the child such as age, gender and prematurity at birth^(5)^. It is noteworthy that the child’s interaction with other people and the social environment is the basis for the development of skills that will continue throughout life^(4)^.

Child development is a process that begins at conception, but the association between pregnancy planning and child development has been little analyzed. In the United Kingdom, a cohort study revealed that children whose mothers reported having unplanned pregnancies had worse cognitive development at three and five years of age, but this association disappeared when adjusted by socioeconomic variables^(9)^. In Brazil, the only research study that investigated this correlation evidenced that it is not planning, but acceptance of the pregnancy that is associated with child development, so that children born to women who did not accept the pregnancy had greater difficulty in language development and fine motor coordination at four years old, when compared to children born to mothers who accepted pregnancy up to the 4^th^ month^(10)^. In another cohort from the United Kingdom, it was also verified that children whose mothers did not want to get pregnant had a lower socio-emotional development score at five years of age^(11)^. In India, seven- and eight-year-olds who were born to women with unintended pregnancies had worse results in the assessment of vocabulary, math and reading skills^(12)^. However, a North American study did not find any association between intention to become pregnant and cognitive or social-emotional child development^(13)^.

It is important to emphasize that the studies cited used different strategies to measure what was called intention to get pregnant, which can limit the comparison and contribute to the inconsistency of the findings. However, there are instruments that incorporate fundamental aspects of planning a pregnancy, which involve desire, intention, partner support and measures related to conception, such as the London Measure of Unplanned Pregnancy^(14)^, which is translated and validated for use in Brazil^(15)^ and in other countries^(16)^. Thus, considering the availability of a reliable instrument for assessing pregnancy planning and that there is controversy regarding the association with child development, this study aimed at analyzing the correlation between child development and pregnancy planning and other associated aspects.

## Method

### Study design

A cross-sectional study that was part of a broader research study entitled “*Programa BEM (Brincar Ensina a Mudar): o brincar na rotina diária para a promoção do desenvolvimento infantil*” [“PTC (Play Teaches to Change) Program: Playing in the daily routine to promote child development”].

### Study locus

The participating families were recruited from Child Education Centers (*Centros de Educação Infantil*, CEIs) from a peripheral district of the municipality of São Paulo, a socially disadvantaged area, with almost 7,000 families living in extreme poverty, more than 15,500 families registered in income distribution programs and more than 15% of pregnant adolescents^(17)^.

### Participants

The research subjects were mothers and their children from 11 to 23 months of age. The study participants were 125 dyads who met the eligibility criteria: children aged between 11 and 23 months old at the time of recruitment, enrolled in the selected CEIs, whose biological mother was responsible for daily care. The exclusion criterion was the child presenting clinical conditions that could interfere with the typical course of child development.

### Study variables

Child Development (CD), a dependent variable of the study, was assessed using the Brazilian version of the Ages & Stages Questionnaire (ASQ-BR)^(18)^, for the evaluation of children aged from six to 60 months old. The instrument consists of 18 questionnaires, one for each age range, and assesses child development in five domains: a) communication, b) broad motor coordination, c) fine motor coordination, d) problem solving, and e) personal/social. For children born prematurely, age adjustment was used^(18)^. All questionnaires have the same structure, consisting of five blocks, one for each domain, with six questions each, so that there is a total of 30 questions at the end. The questions are specific to evaluate certain activity, with three answer options: “yes”, if the child is able to perform the activity every time; “sometimes”, when the child is not always able to perform the activity successfully; “not yet” when the child is unable or has never performed the activity^(18)^. For each domain, the child obtains a score between 0 and 60, which is classified by age group as “adequate child development” or “inadequate child development”. ASQ-BR has adequate internal consistency; the different questionnaires used in the research for each age group presented Cronbach’s Alpha values ≥ 0.60.

Pregnancy planning, the main independent variable, was measured using the Brazilian version of the London Measure of Unplanned Pregnancy (LMUP)^(15)^. It is an instrument composed of six questions that refer to the use of contraceptives, motherhood context, intention, desire to have a baby, discussion with the partner and preconception preparation. For each of the questions, the answers can vary from 0 to 2 points, so that, with the sum of the points, it is possible to classify the pregnancy as planned (10-12 points); ambivalent (4-9 points) or unplanned (0-3 points). LMUP presents adequate internal consistency with Cronbach’s alpha = 0.813.

The covariates referred to the *socioeconomic characteristics*: monthly family income range (up to 1 minimum wage; between 1 and 3 minimum wages; more than 3 minimum wages), receiving Family Grant (*Bolsa Família*) (yes/no), marital status (with/without partner), mother’s schooling in years of study (5-9; 10-12; ≥13) and maternal work (employed/unemployed); to the *maternal characteristics*: age at childbirth in years old (≤19; 20-29; ≥30), number of children (1; >1), prenatal care (yes/no), and number of prenatal consultations (<6; ≥6); and to the *child’s characteristics*: child’s age in months old (≤12; 13-15; 16-18; ≥19), gender (male/female), and prematurity at birth (yes/no). *Bolsa Família* refers to the Brazilian governmental program to fight against poverty and inequality, which includes an income supplement as one of its benefits^(19)^. The number of prenatal care consultations was defined as recommended by the Ministry of Health, which considers 6 as the minimum number of consultations for adequate prenatal care; and prematurity at birth was defined for children who were born before completing 37 gestational weeks^(20)^.

### Data collection

Data collection was carried out through previously scheduled interviews, carried out at the women’s homes, with a mean duration of 1h30min, in the period from July 2019 to March 2020, by trained interviewers, professionals and undergraduate students in the health area, who used *tablets* with forms inserted in the REDCap (Research Eletronic Data Capture) software.

### Data analysis

For data analysis, the Stata software, version 15.0, was used. The results were described using absolute and relative frequencies, means (x) and standard deviations (SD). Child development and pregnancy planning were analyzed as continuous quantitative variables, considering that the higher the final score, the better the child development (0-60 points), just as the higher the final score, the higher the pregnancy planning (0-12 points). Normal distribution of the variables was verified using the Shapiro-Wilk test, adopting a significance level of 5%. The dependent (child development) and independent (pregnancy planning) variables did not present normal distribution; therefore, non-parametric tests for independent samples were used, namely: Mann-Whitney (comparison of means for qualitative variables with two categories) and Kruskal-Wallis (comparison of means for qualitative variables with three or more categories). The Spearman’s correlation test was also used to assess the association between the dependent and independent variables. The magnitude of the correlation coefficient was interpreted as strong when the value was equal to or greater than 0.8; as moderate when the value was between 0.6 and 0.7; as reasonable when the value was between 0.3 and 0.5; and as weak when the value was less than 0.3^(21)^. The significance level adopted was 5%.

### Ethical aspects

The broader research project was approved by the Research Ethics Committee of the José Luiz Egydio Setúbal Foundation, under substantiated opinion number 3,448,089.

## Results

Table 1 presents the socioeconomic, maternal and child’s characteristics. More than half of the families had monthly incomes of around 1 to 3 minimum wages (R$ 1,000.00-R$ 3,000.00); one-fifth received *Bolsa Família*, three-quarters of the women had a partner, two-thirds were employed, had a mean of 12.2 years of study, at the birth of the child studied their mean age was 28.7 years old, more than half had more than one child, almost all attended prenatal care and, of these, the vast majority had six or more visits. The mean age of the children was 16.2 months old, more than half were male and 8.8% were premature.

**Table 1:** Socioeconomic, maternal and child’s characteristics of the participants (n=125). São Paulo, SP, Brazil, 2020

Variables	n	%
*Socioeconomic*		
Monthly family income (in R$)		
Up to 1 minimum wage*	31	24.8
Between 1 and 3 minimum wages	69	55.2
More than 3 minimum wages	25	20.0
Receives *Bolsa Família*		
Yes	24	19.2
No	101	80.8
Marital status		
Has a partner	93	74.4
No partner	32	25.6
Maternal schooling (years of study) *x* (SD)^†^	12.2 (3.0)	
5-9	26	20.8
10-12	51	40.8
≥13	48	38.4
Maternal work		
Employed	81	64.8
Unemployed	44	35.2
*Maternal*		
Age at birth of the child (years old) *x* (SD)	28.7 (7.2)	
≤19	14	11.2
20-29	57	45.6
≥30	54	43.2
Number of children *x* (SD)	1.9 (1.2)	
1	57	45.6
>1	68	54.4
Attended prenatal care		
Yes	123	98.4
No	2	1.6
Number of prenatal consultations^‡^ *x* (SD)	9.9 (3.8)	
<6	13	11.3
≥6	102	88.7
*Child*		
Age (months old) *x* (SD)	16.2 (3.4)	
≤12	23	18.4
13-15	28	22.4
16-18	36	28.8
≥19	38	30.4
Gender		
Male	69	55.2
Female	56	44.8
Prematurity		
Yes	11	8.8
No	114	91.2

* Current minimum wage = R$ 1,045.00, Brazil, 2020;

† x (SD) = Mean (Standard Deviation);

‡ Data were not obtained for the entire sample

With regard to pregnancy planning (Table 2), 17.6% of the women did not plan the pregnancy; a quarter had planned it and more than half of the pregnancies were classified as ambivalent. As for child development, more than 60% of the children had adequate development in all domains (adequate CD), with a higher percentage in the “personal/social” domain (79.2%) and a lower percentage in the “fine motor coordination” domain (60.0%).

**Table 2:** Distribution of pregnancy planning and child development (n=125). São Paulo, SP, Brazil, 2020

Variables	n	%
*Pregnancy planning x (SD)* *	6.6 (3.1)	
Not planned	22	17.6
Planned	31	24.8
Ambivalent	72	57.6
*Child development*		
Communication *x* (SD)	41.4 (14.2)	
Adequate	86	68.8
Inadequate	39	31.2
Broad motor coordination *x* (SD)	49.0 (14.0)	
Adequate	95	76.0
Inadequate	30	24.0
Fine motor coordination *x* (SD)	45.0 (12.5)	
Adequate	75	60.0
Inadequate	50	40.0
Problem solving *x* (SD)	44.1 (11.7)	
Adequate	92	73.6
Inadequate	33	26.4
Personal/social *x* (SD)	46.9 (10.8)	
Adequate	99	79.2
Inadequate	26	20.8

* *x* (SD) = Mean (Standard Deviation)

The analysis of the pregnancy planning scores according to the socioeconomic, maternal and child’s characteristics (Table 3) showed that mothers who did not receive *Bolsa Família* had a significantly higher pregnancy planning mean score (p=0.0271), as well as mothers with a partner (p=0.0013) and who attended 6 or more prenatal consultations (p=0.0230). There was a positive correlation between pregnancy planning and maternal schooling (p=0.0037) and mother’s age at childbirth (p=0.0004) and a negative correlation with the number of children (0.0380).

**Table 3:** Distribution of the pregnancy planning scores according to socioeconomic, maternal and child variables (n=125). São Paulo, SP, Brazil, 2020

Categorical variables	Pregnancy planning	
	*x* (SD)^†^	p
*Socioeconomic*		
Monthly family income		0.2909
Up to 1 minimum wage*	5.9 (2.8)	
Between 1 and 3 minimum wages	6.7 (3.1)	
More than 3 minimum wages	7.2 (3.2)	
Receives *Bolsa Família*		**0.0271**
Yes	5.3 (2.6)	
No	6.9 (3.1)	
Marital status		**0.0013**
Has a partner	7.1 (3.0)	
No partner	5.1 (2.8)	
Work		0.3669
Employed	6.8 (3.0)	
Unemployed	6.2 (3.0)	
*Maternal*		
Attended prenatal care		0.0600
Yes	6.7 (3.0)	
No	3.0 (0,0	
Number of prenatal consultations^‡^		**0.0230**
<6	4.9 (2.3)	
≥6	7.0 (3.1)	
*Child*		
Gender		0.2622
Male	6.9 (2.9)	
Female	6.3 (3.2)	
Prematurity		0.4250
Yes	7.4 (3.8)	
No	6.5 (3.0)	
Continuous variables	*R*	*p*
Mother’s schooling (years of study)	0.2581	**0.0037**
Mother’s age at childbirth (years old)	0.3146	**0.0004**
Number of children	-0.1858	**0.0380**
Child’s age (months old)	0.0191	0.8324

* Current minimum wage = R$ 1,045.00, Brazil, 2020;

† x (SD) = Mean (Standard Deviation);

‡ Data were not obtained for the entire sample

The scores of all the child development domains according to socioeconomic, maternal and child variables are presented in Table 4. In the “communication” domain, children from families receiving *Bolsa Família* and female children presented a significantly higher mean value (p<0.05). There was a positive correlation between the “broad motor coordination” and “fine motor coordination” domains with child’s age (p<0.05). The “problem solving” domain showed no association with any of the variables analyzed and, in the “personal/social” domain, female children presented a significantly higher mean value (p<0.05) and there was a positive correlation of this variable with child’s age (p=0.0014).

**Table 4:** Distribution of the child development domain scores according to socioeconomic, maternal and child variables (n=125). São Paulo, SP, Brazil. 2020

Categorical variables	Child development domains									
	Communication		Broad motor coordination		Fine motor coordination		Problem resolution		Personal/Social	
	*x* (SD)^†^	p	*x* (SD)	p	*x* (SD)	p	*x* (SD)	p	*x* (SD)	p
*Socioeconomic*										
Monthly family income (R$)		0.4136		0.7441		0.2686		0.2804		0.3549
Up to 1 minimum wage*	38.2 (15.3)		48.4 (15.6)		43.1 (13.3)		41.1 (12.6)		44.5 (10.8)	
Between 1 and 3 minimum wages	42.1 (13.8)		48.6 (13.6)		44.9 (11.9)		45.0 (11.3)		47.9 (10.6)	
More than 3 minimum wages	43.2 (14.1)		50.6 (12.6)		48.0 (13.0)		45.4 (11.6)		47.0 (11.1)	
Receives *Bolsa Família*		**0.0094**		0.8220		0.4714		0.0882		0.2035
Yes	47.3 (14.4)		48.5 (16.2)		46.7 (11.9)		48.1 (8.8)		49.4 (10.1)	
No	40.1 (13.9)		49.1 (13.3)		44.6 (12.6)		43.2 (12.2)		46.3 (10.9)	
Marital status		0.4699		0.3100		0.1371		0.7696		0.4156
Has a partner	42.0 (13.8)		50.1 (12.5)		46.2 (11.5)		44.0 (11.7)		47.4 (10.5)	
No partner	39.5 (15.4)		45.8 (17.0)		41.7 (14.7)		44.5 (11.9)		45.3 (11.7)	
Maternal work		0.6089		0.4152		0.3815		0.2060		0.3871
Employed	40.9 (14.8)		49.5 (13.3)		45.5 (13.0)		45.0 (12.0)		46.4 (10.4)	
Unemployed	42.3 (13.3)		48.0 (14.8)		44.2 (11.5)		42.5 (11.2)		47.7 (11.6)	
*Maternal*										
Attended prenatal care		0.4153		0.3013		0.9601		0.1193		0.0523
Yes	41.5 (14.3)		49.0 (13.9)		45.0 (12.4)		44.4 (11.6)		46.6 (10.7)	
No	35.0 (7.1)		45.0 (7.1)		45.0 (21.2)		30.0 (14.1)		60.0 (0.0)	
Number of prenatal consultations^‡^		0.8132		0.2847		0.0696		0.1307		0.8894
<6	40.4 (15.5)		43.8 (18.7)		39.2 (12.9)		49.2 (8.6)		47.3 (9.0)	
≥6	41.7 (14.3)		49.6 (13.0)		45.8 (12.4)		43.9 (11.4)		46.2 (11.1)	
*Child*										
Gender		**0.0109**		0.0845		0.8499		0.3208		**0.0189**
Male	38.2 (15.4)		47.5 (14.5)		44.4 (13.6)		43.1 (12.3)		44.8 (11.2)	
Female	45.3 (11.7)		50.8 (12.8)		45.8 (10.9)		45.4 (11.0)		49.5 (9.8)	
Prematurity		0.2035		0.9278		0.8908		0.5070		0.3209
Yes	35.9 (16.1)		45.0 (21.3)		45.0 (13.0)		40.9 (16.2)		50.0 (10.0)	
No	41.9 (14.0)		49.3 (13.0)		45.0 (12.5)		44.4 (11.3)		46.6 (10.8)	
Continuous variables	*r*	*p*	*r*	*p*	*r*	*p*	*r*	*p*	*r*	*p*
Mother’s schooling (years)	0.0212	0.8145	-0.0613	0.4974	0.1319	0.1426	0.1265	0.1598	-0.0742	0.4112
Mother’s age at childbirth (years old)	-0.0519	0.5656	0.1102	0.2211	0.0394	0.6630	-0.0301	0.7391	-0.1463	0.1035
Number of children	-0.0895	0.3209	-0.0187	0.8363	-0.0448	0.6195	0.0064	0.9440	-0.1347	0.1342
Child’s age (months old)	-0.1487	0.0980	0.3777	**<0.001**	0.2271	**0.0109**	-0.1480	0.0996	0.2836	**0.0014**

* Current minimum wage = R$ 1,045.00, Brazil, 2020;

† x (SD) = Mean (Standard Deviation);

‡ Data were not obtained for the entire sample

The correlation analysis between the pregnancy planning score and the child development domains shows that there was no statistically significant association between these variables (p>0.05).

**Table 5:** Correlation between the pregnancy planning score and the child development domains. São Paulo, SP, Brazil, 2020

Child development domains	Pregnancy planning	
	r	p
Communication	-0.0430	0.6339
Broad motor coordination	-0.0038	0.9661
Fine motor coordination	0.0641	0.4775
Problem solving	-0.0839	0.3525
Personal/Social	0.0203	0.8220

## Discussion

This study evaluated pregnancy planning and child development in children aged from 11 to 23 months old and found no correlation between these variables. This finding confirms results found in the few studies that evaluated such correlation in the United Kingdom^(9)^ and in Brazil^(10)^.

Pregnancy planning and child development are topics of particular interest to Nursing, considering their prominent role in maternal and child health care, especially in primary care. When it comes to children’s health, the monitoring of child development is a priority and cross-cutting action among the actions developed by Nursing professionals, which is initiated in the preconception period, intensifies in prenatal care and extends to the childcare Nursing consultations. Thus, the study results contribute to the Nursing practice by providing knowledge to support care longitudinality and the promotion of child development before, during and after pregnancy.

It is noteworthy that this study used a validated instrument to assess pregnancy planning^(14)^, which also considers important issues in the dynamics of the intention to get pregnant, such as the relationship with the partner and the ambivalences, considering that the woman/couple is not always able to clearly express their reproductive desires and intentions^(22)^. Thus, given the lack of uniformity in the terms and measures used to assess pregnancy planning in several studies, it is considered that the results of this study represent an advance for using an instrument specifically developed to assess pregnancy planning, translated and validated for the Brazilian context^(15)^.

The results obtained regarding pregnancy planning showed that only 25% of the pregnancies had been planned, a proportion that is lower than the 33% found among women evaluated at the time they sought the health services to confirm their pregnancies and had a positive result^(23)^; and it was especially associated with socioeconomic variables, not receiving *Bolsa Família*, marital status and mother’s schooling, in accordance with the findings of other studies^(23-24)^ and consistent with the evidence that unplanned and unintended pregnancies are more frequent in contexts of greater social and economic disadvantage^(25)^.

The low proportion of planned pregnancies indicates the need for interventions aimed at increasing their occurrence. In this sense, Nursing professionals play an important role because health promotion spaces in primary care such as contraceptive consultations, cervical cytology collection, postnatal consultations and activities with adolescents in schools, represent a valuable opportunity to provide adequate contraceptive and preconception care^(26)^. In addition, these interventions should be aimed mainly at women from the most disadvantaged social groups^(27)^, as highlighted by the results obtained.

Not having found any association between pregnancy planning and child development is a significantly favorable result, as it indicates that, although the number of children born to women whose pregnancy was classified as unplanned is high, this condition does not interfere with the child development. It is possible to conjecture that children whose mothers did not plan their pregnancies have the same chances of reaching their developmental potential when compared to those born to women who planned their pregnancies.

In this context, the discussion is whether the effects of unplanned pregnancy on obstetric and child outcomes described in the literature do not come from another element, which would be acceptance of the pregnancy, as evidenced in a Brazilian study^(10)^. Thus, another study^(28)^ urges the scholars to rethink whether it is really pregnancy planning, that is, something that occurs before conception, that produces any adverse outcome of this pregnancy, whether during prenatal care, at delivery or after birth, or whether it is the fact that the woman/couple accepts an ongoing pregnancy, regardless of whether it is planned or not. This is because, by accepting pregnancy, women and couples are able to adopt behaviors and take steps to ensure that the pregnancy is healthy. Therefore, it is fundamental that Nursing interventions in the prenatal period include special care in the monitoring of families who did not plan the pregnancy, especially those who did not accept it, with a view to favoring obstetric and child outcomes.

It is also important to point out that there are questions about the validity of the information about the intention to become pregnant when obtained after birth, justified by the fact that mothers and fathers get involved with their children and start to consider pregnancy as desired, so that the pregnancy intention measure can have some error and estimates of its effect on child development can be biased^(13)^. Anyway, even if the pregnancy was not intentional, if the parents get involved with it, they may start to consider it as desired, which would provide a positive involvement in the development of their children.

The proportion of children with inadequate development, in the different domains evaluated, corroborates previous results for the Brazilian population, which found percentages between 17% and 30% of children with probable developmental delay^(29)^. In addition, they reiterate global estimates that, in low- and middle-income countries, 43% of the children under the age of five are at risk of not reaching their maximum development potential due to extreme poverty^(30)^. The high percentage of children with inadequate development draws the attention to the need to continue investing in the promotion of child development, with actions that favor and encourage the establishment of strong and lasting adult-child interactions.

Interventions aimed at promoting positive parenting are promising tools for improving the parenting practices and child development in low- and middle-income countries^(31)^. Therefore, it is crucial that health professionals, mainly Nursing professionals, are trained to monitor the families throughout the process of preparation for the baby’s arrival and, subsequently, be able to recognize risk situations for the development of the child. In primary health care, Nursing professionals stand out for the privileged position of contact they maintain with children and families, especially during the first years of life^(32)^, when the foundations are laid for adequate brain development. Thus, Nursing professionals should take advantage of the childcare consultation to guide the families on the importance of the adult-child interaction, with involvement in daily activities, in order to contribute to the children attaining their maximum development potential.

Child development was mainly associated with the child’s variables, as two domains were associated with the child’s gender and three showed a positive correlation with age, despite consistent evidence pointing to a significant influence of the social and economic context on the development of individuals, since the first years of life^(33)^. In this study, there was only a statistically significant association with a socioeconomic variable, that is, the highest mean of the communication domain among children from families that are beneficiaries of the *Bolsa Família* Program. This finding is of special interest, as it seems to suggest a positive contribution of this income transfer program to child development for families in extreme poverty. Surveys that evaluated the impact of income transfer programs focused especially on children’s nutrition: a review that included nine primary research studies and two literature reviews showed positive results of the *Bolsa Família* Program on food safety, food intake and children’s anthropometric indicators^(34)^; a research study that evaluated the impact of the *Bolsa Família* Program on children’s schooling showed that the program increased girls’ participation at school^(35)^. Not having observed any association between child development and other socioeconomic conditions in this study could be explained by the fact that the families present very similar characteristics, as the children attended early childhood education centers located in socially disadvantaged areas.

The following stand out as limitations of this study: the fact that the families were recruited in only one district of the municipality of São Paulo and that data on pregnancy planning occurred from 11 to 23 months after birth of the child, which may have imposed memory bias, although the LMUP has proven to be a stable instrument. In addition, as this is a cross-sectional study, it is not possible to determine the cause-effect correlation of the results presented, which leads to the need to conduct longitudinal studies.

Despite these limitations, our results contribute to expanding knowledge, which is currently scarce, in relation to the subject matter studied. In addition to that, this is a pioneering study that analyzed the association between child development and pregnancy planning, using an instrument validated for use in Brazil^(15)^. For future studies, it is interesting to investigate the bond or quality of interaction between mother and child, according to the classification of pregnancy planning (unplanned, ambivalent and planned), and what influence they can exert on child development, seeking evidence that allows confirming the hypothesis that interaction and bonding since the prenatal period influence child development more than the condition of pregnancy planning. As this study only included children aged between 11 and 23 months old, the inclusion of other age groups could be considered in future research studies.

## Conclusion

There was no correlation between pregnancy planning and child development in children living in socially disadvantaged areas; therefore, pregnancy planning does not interfere with child development in children aged from 11 to 23 months old, but the low frequency of planned pregnancies and the high percentages of inadequate child development show the need to invest in training health professionals, both for contraception and preconception care, and for the promotion of child development, especially in socioeconomically disadvantaged contexts.
